# Dosimetric impact of using a commercial metal artifact reduction tool in carbon ion therapy in patients with hip prostheses

**DOI:** 10.1002/acm2.13314

**Published:** 2021-06-23

**Authors:** Jingfang Zhao, Weiwei Wang, Kambiz Shahnaz, Xianwei Wu, Jingfang Mao, Ping Li, Qing Zhang

**Affiliations:** ^1^ Department of Medical Physics Shanghai Proton and Heavy Ion Center, Fudan University Cancer Hospital Shanghai China; ^2^ Shanghai Key Laboratory of Radiation Oncology Shanghai China; ^3^ Shanghai Engineering Research Center of Proton and Heavy Ion Radiation Therapy Shanghai China; ^4^ Department of Medical physics Shanghai Proton and Heavy Ion Center Shanghai China; ^5^ Department of Radiation Oncology Shanghai Proton and Heavy Ion Center Shanghai China

**Keywords:** carbon‐ion radiotherapy, hip prosthesis, iMAR, metal artifact reduction, prostate cancer, sarcoma

## Abstract

The study investigated the dosimetric impact of an iterative metal artifact reduction (iMAR) tool on carbon ion therapy for pelvic cancer patients with hip prostheses. An anthropomorphic pelvic phantom with unilateral and bilateral hip prostheses was used to simulate pelvic cancer patients with metal implants. The raw data obtained from phantom CT scanning were reconstructed with a regular filtered back projection (FBP) algorithm and then corrected with iMAR. The phantom without hip prosthesis was also scanned and used as a reference ground truth (GT). The CT images of three prostate and four sarcoma patients with unilateral hip prosthesis were also reconstructed by FBP and iMAR algorithm and compared. iMAR algorithm reduced the metal artifacts and the maximum WEPL deviation in phantom images from −19.1 to −0.4 mm. However, the CT numbers cannot be retrieved using iMAR for periprosthetic bone materials, eventually leading to a WEPL deviation of −3.6 mm. The use of iMAR improved large discrepancies in DVHs of PTVs and the gamma index between FBP and GT images but increased the difference in the bladder DVH for bilateral hip prostheses due to newly introduced artifacts. In the patient study, the discrepancies of dose distribution were small on iMAR images when compared with FBP images for most cases, except for two sarcoma cases where gamma analysis failed and dose coverage in 98% of the PTV maximally reduced due to large volume of dark metal artifacts. iMAR reduced the metal artifacts and improved dose distribution accuracy in carbon ion radiotherapy for pelvic cancer. However, the residual and newly introduced artifacts, especially with bilateral hip prostheses, may potentially increase WEPL inaccuracy and dose uncertainty. The use of iMAR has the potential to improve carbon ion treatment planning of pelvic cancer but should be used with caution.

## 1 INTRODUCTION

As the use of hip prostheses is becoming increasingly common in the aging population, the number of these patients in need of radiotherapy is expected to increase.^1^, ^2^ These prostheses are often made using materials with high atomic numbers (*Z*) such as cobalt‐chromium‐molybdenum (Co‐Cr‐Mo) alloy and titanium (Ti) and can cause serious metal artifacts, which not only impairs the visualization of the anatomical structures but also results in inaccurate quantification of the computed tomography (CT) numbers needed for dose calculations. The range of particle beam is predicted by mapping the CT numbers to the relative linear stopping power (RLSP) of heterogeneous tissues. As most of the dose is delivered at a specific depth at the end of range known as the Bragg peak, metal artifacts may cause uncertainties in the range calculation, eventually shifting the position of Bragg peak. Furthermore, in contrast to photon therapy with a relatively shallow decrease of dose with depth, particle radiotherapy with a rapid Bragg peak distal fall‐off could be more sensitive to variations in CT numbers caused by metal artifacts.^3^, ^4^ This could potentially compromise the tumor coverage and lead to unnecessary dose to surrounding normal tissue.

Carbon ion radiotherapy provides a higher biologically effective dose when compared to photon and proton radiotherapy.^5^ Multiple studies reported excellent disease control with low toxicity levels when using carbon ion radiotherapy for pelvic cancer.^6^, ^7^, ^8^, ^9^ Several pelvic cancer patients, including some with hip prostheses, were treated with carbon ions at our facility. However, due to the considerable size of the prostheses, very pronounced artifacts were produced in the planning CT images leading to uncertainty in the calculation of radiation doses.

To overcome this problem, the metal artifacts can be reassigned manually either by CT numbers or the relative linear stopping power (RLSP).^10^ However, this technique is very time consuming, and its accuracy depends on the experience of the dosimetrist. Using a combination of megavoltage (MV) and kilovoltage (kV), CT imaging has been shown to improve both contouring and dose calculation accuracy. However, MVCT is not widely available, and it significantly increases the radiation dose.^11^

Iterative metal artifact reduction (iMAR) algorithm (Siemens Healthcare, Forchheim, Germany) has been proposed for clinical use in diagnostic imaging to reduce the visual conspicuity of metal artifacts and improve the contouring accuracy of the target volume and critical organs.^12^, ^13^, ^14^, ^15^, ^16^ Very few investigations have been performed to evaluate its dosimetric benefit as the planning CT images on particle treatment planning with various metal implants. Axente et al.^17^ performed a limited evaluation on the impact of iMAR on proton therapy by using an electron density phantom with metallic rods and found that proton beam range errors could be reduced with iMAR. However, further studies are needed to comprehensively evaluate the impact of iMAR in proton planning. Andersson^18^ found that dose distribution differences in the areas of most severe artifacts were reduced by iMAR in head and neck phantom but not all. And more clinical studies using different implants are necessary.

To our knowledge, there has not been any systematic and quantitative evaluation on using iMAR images for carbon ion treatment planning of pelvic cancer with hip prostheses. Therefore, this study aimed to investigate the dosimetric impact of the iMAR algorithm by using both an anthropomorphic phantom and patient cases in terms of CT numbers, water equivalent path length (WEPL), and dose distributions.

## 2 METHODS AND MATERIALS

### 2.1 Development of the iMAR algorithm

iMAR algorithm^19^ reduces the metal artifacts on images by iteratively applying the normalized metal artifact reduction (NMAR) tool and frequency split metal artifact reduction (FSMAR) tool several times. NMAR^20^ is a sinogram in‐painting based method with a generalized normalization technique. It was developed to reduce metal artifacts and prevent the introduction of new artifacts. FSMAR^21^ combines the high frequencies of an original uncorrected image with the low frequencies of an image that was corrected by a sinogram interpolation‐based metal artifact reduction method. FSMAR can create an image with clear edges and fine anatomical details. The iMAR algorithm used the final NMAR images as the input to the FSMAR and then obtained the FSMAR‐corrected images to feed the next iteration. The iterative numbers, Hounsfield Unit (HU) thresholds for segmentation, and filter parameters were set and modeled in the algorithms according to different metal implants such as the hip prosthetic implant, dental implants, and lung coil. The algorithms are pre‐set and user‐selectable depending on the type of implants.

### 2.2 Anthropomorphic phantom study

#### 2.2.1 Phantom imaging

**Fig. 1:**
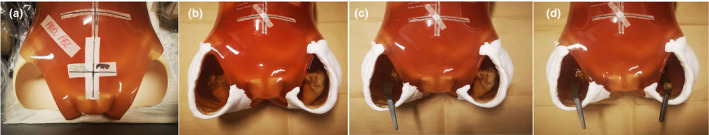
Anthropomorphic phantom used in the study: (a) pelvic anthropomorphic phantom before reconstruction. (b) Modified pelvic anthropomorphic phantom as ground truth. (c) Modified phantom with a unilateral hip prosthesis. (d) Modified phantom with bilateral hip prostheses.

Phantom studies were conducted on the pelvic part of a whole‐body anthropomorphic phantom (PBU‐60, KYOTO KAGAKU Co. LTD) [Fig. 1(a)]. Two swine femoral heads and home‐made gelatin gel were used as human tissue substitutes to simulate normal patients without metal implants. The phantom was first scanned, and the images were used as the artifact‐free ground truth (GT) [Fig. 1(b)]. Subsequently, the Co‐Cr‐Mo alloy hip prostheses with Ti alloy shell were used to replace one side and then both of the swine femur heads to simulate patients with unilateral [Fig. 1(c)] or bilateral hip prostheses [Fig. 1(d)]. The phantom was scanned with the same CT protocol used for CT calibration. The obtained raw data were reconstructed using the filter back projection (FBP) algorithm and then corrected with iMAR. For the iMAR reconstruction, the hip implant pre‐set was selected.

#### 2.2.2 CT calibration

A CT calibration curve was generated by using the water equivalent method.^22^, ^23^ The curve helped to convert CT numbers to RLSP, used for evaluation of the range and dosimetric impact with and without the iMAR correction. The relationship between CT numbers and RLSP (relative to water) was established with five linear fits and six control points. The first five points were 0 at HU of −1,000, 0.8 at HU of −200, 0.95 at HU of −100, 1.0 at HU of 0, and 1.05 at HU of 50. The last point was confirmed as 2.602 at HU = 3,095, which was calculated according to measured CT numbers of aluminum in a standard phantom CT image. The in‐house developed standard phantom with water and an aluminum rod was scanned by using the pelvic protocol on a Siemens SOMATOM Definition AS+ CT (Siemens Healthcare, Forchheim, Germany). The scanning parameters used were as follows: a tube voltage of 120 kVp, tube current of 300 mAs, a field of view of 500 mm, a B30s reconstruction kernel, and 2‐mm‐thick slices. The linear fitting of the RLSP was calculated as a function of the CT numbers for the six control points.

#### 2.2.3 Beam range evaluation

To quantify the range differences based on the iMAR, FBP, and GT images, the WEPL was calculated as follows:

(1) $$\text{WEPL}=L_{w}=L_{m}\times \text{RLSP}$$

where *L_w_* and *L_m_* are the water equivalent length and the physical length in the medium, respectively. RLSP can be calculated with the CT numbers by the CT calibration curve.

WEPLs were first calculated on the FBP reconstruction CT slice with the most prominent artifacts and then by using the corresponding path on the iMAR and GT images. The differences in WEPL were compared between FBP/iMAR images and GT images.

(2) $$\Delta \text{WEPL}={\text{WEPL}}_{\text{iMAR}/\text{FBP}}‐{\text{WEPL}}_{\text{GT}}$$

#### 2.2.4 Dosimetric evaluation

The dosimetric evaluation was conducted by comparing optimized plans on FBP, iMAR, and GT images. In order to observe the impact of artifacts on tumors at various locations, prostate cancer and sarcoma cases were selected since they were the most common cases with hip prostheses treated in our hospital. The planning target volume (PTV) and critical organs (bladder and rectum) were delineated on the GT images by a radiation oncologist and then copied to FBP and iMAR images after registration with GT images. The air in the rectum was overridden with RLSP as water, as per departmental protocol. Treatment plans were first optimized on the FBP and iMAR images and then recalculated on GT images. All plans were generated by the Syngo treatment planning system (VB13, Siemens health solution, Erlangen, Germany) with a pencil beam algorithm. The beam directions were chosen to avoid passing through the metal at the beam path. For cases with a unilateral hip prosthesis, lateral and oblique beam angles were chosen, while for cases with bilateral hip prostheses, only the anterior to posterior vertical beam could be used.

Variations in the 3D global dose distributions were analyzed with gamma analysis (γ < 1, 1% dose difference/2 mm distance to agreement) using the PTW Verisoft 7.1 software (PTW, Freiburg, Germany) with a local difference setting and a cutoff dose of 10% of the maximum dose of calculated volume. In addition, the gamma analysis of 2D planar dose distribution was also performed on each slice to evaluate the effect of artifacts on regional dose distribution, and the worst results were recorded. The DVH was used to evaluate the dose received by the PTV. The DVH analysis of PTVs included D_V98%_ (percentage of prescription dose covering 98% of the PTV), V_D95%_ (percentage of PTV volume covered by 95% of prescription dose), D_1cc_ (the percentage of the prescription dose received by 1cc of the PTV volume), and D_mean_ (mean dose). For critical organs (bladder and rectum), D_mean_ and V_D50 Gy(RBE)_ (the relative volume receiving 50 Gy [RBE]) were compared. V_D50 Gy(RBE)_ is a significant risk factor for the occurrence of late gastrointestinal toxicity in prostate cancer^24^ and has been chosen for comparison. For the sarcoma case, the rectum and the bladder were too far away from the target to get a significant radiation dose and were, therefore, not evaluated.

### 2.3 Patient study

#### 2.3.1 Image acquisition

CT Images from seven patients with unilateral metal hip prosthesis were acquired using the same CT protocols as per the phantom study. All the CT data of patients were reconstructed with FBP and then corrected with the iMAR algorithm using the hip implant pre‐set setting. The present study was approved by the local hospital ethics committee.

#### 2.3.2 Quantitative image analysis

On each set of the patient's images, four regions of interest (ROIs) were predefined on five consecutive slices on the FBP reconstructed CT images and then copied on the iMAR reconstructed images. The four ROIs were positioned on a dark streak near the metal implant (ROI‐D metal), a bright streak near the metal implant (ROI‐B metal), a dark or bright streak on fatty tissue (ROI‐fat), and all the bone material at the ipsilateral side of the metal (ROI‐bone). The CT numbers and noise (standard deviation of CT numbers) of all the four ROIs were compared between FBP and iMAR images. The corresponding reference CT numbers within the ROIs were determined on the same tissue, which was not affected by metal artifacts on the same image set. The positions of the reference ROIs were determined individually according to the anatomy of every patient using a 12‐mm‐diameter cursor on each slice.

#### 2.3.3 Dosimetric comparison

Information about implants and disease characteristics of the seven patients is summarized in Table 1. Patient plans were optimized on the FBP images and then recalculated on the iMAR images with identical beam parameters. Techniques used in the patient treatment plans were the same as in the phantom study. The DVH parameters used to evaluate the phantom were also used to compare dosimetric differences between the FBP and iMAR reconstructed images.

**Table 1:** Patient data about metal implants, location of implants, and indications.

Patient	Metal implant	Location	Indication
1	Femur head and stem	Right	Osteosarcoma
2	Femur head, stem, and hip bone	Left	Osteosarcoma
3	Femur head and stem	Right	Chondrosarcoma
4	Femur head and stem	Left	Chondrosarcoma
5	Stem	Left	Prostate cancer
6	Femur head	Left	Prostate cancer
7	Femur head and stem	Left	Prostate cancer

## 3 RESULTS

### 3.1 Anthropomorphic phantom study

#### 3.1.1 Phantom images

**Fig. 2:**
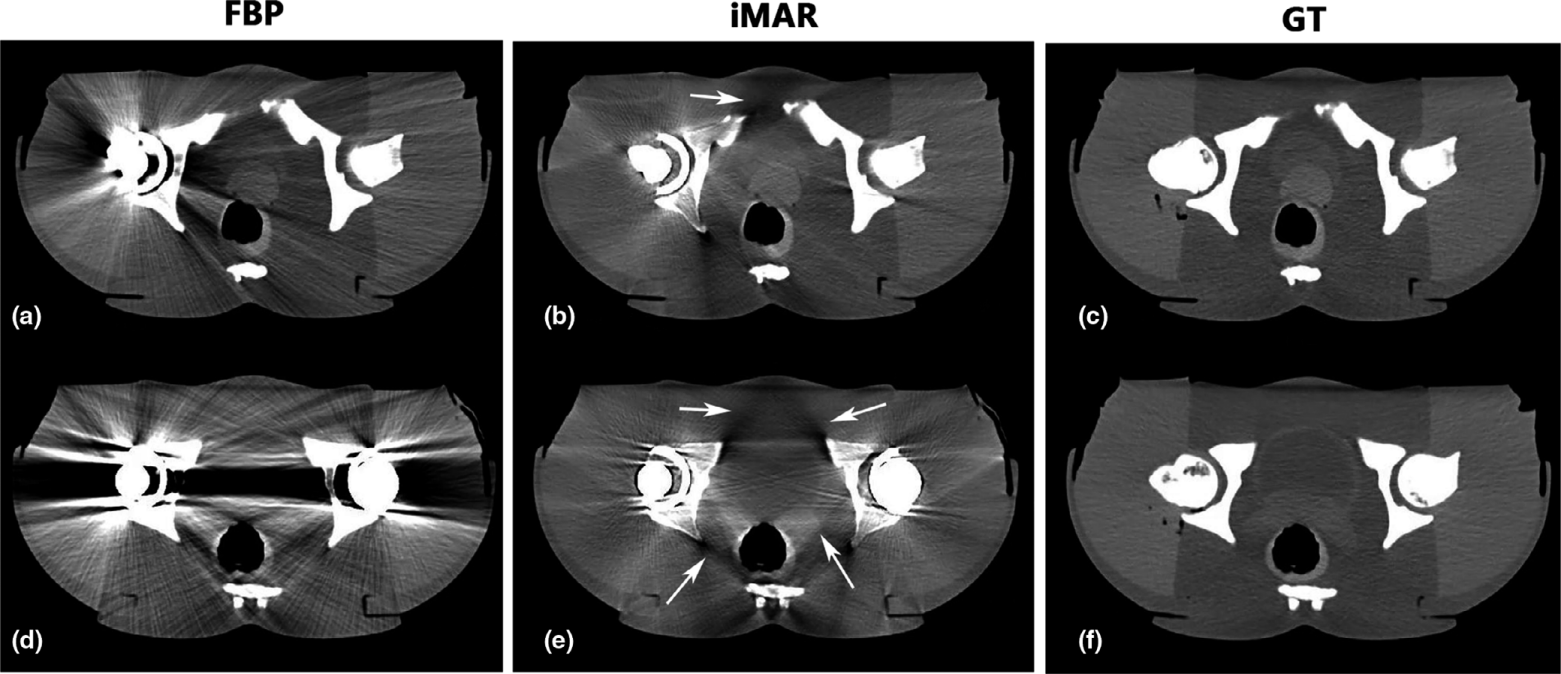
CT images of pelvic phantom: FBP and iMAT CT images of a pelvic phantom with unilateral (a and b) and bilateral (d and e) hip‐prostheses compared with an FBP image without metal implants serving as a reference ground truth (c and f). The arrows illustrate the newly generated artifacts adjacent to the periprosthetic bone in iMAR images (Window level: 40 HU and window width: 400 HU).

Figure 2 illustrates the CT images obtained from the phantom with unilateral or bilateral hip‐prostheses before and after iMAR correction, together with GT images. Visual inspection of these images showed that the iMAR algorithm significantly reduced the metal artifacts. However, new artifacts were introduced, especially in the bilateral hip prosthesis case. Most of the newly generated artifacts were located at the periprosthetic bones and on soft tissue adjacent to bony structures.

#### 3.1.2 CT number profile and WEPL calculation

**Fig. 3:**
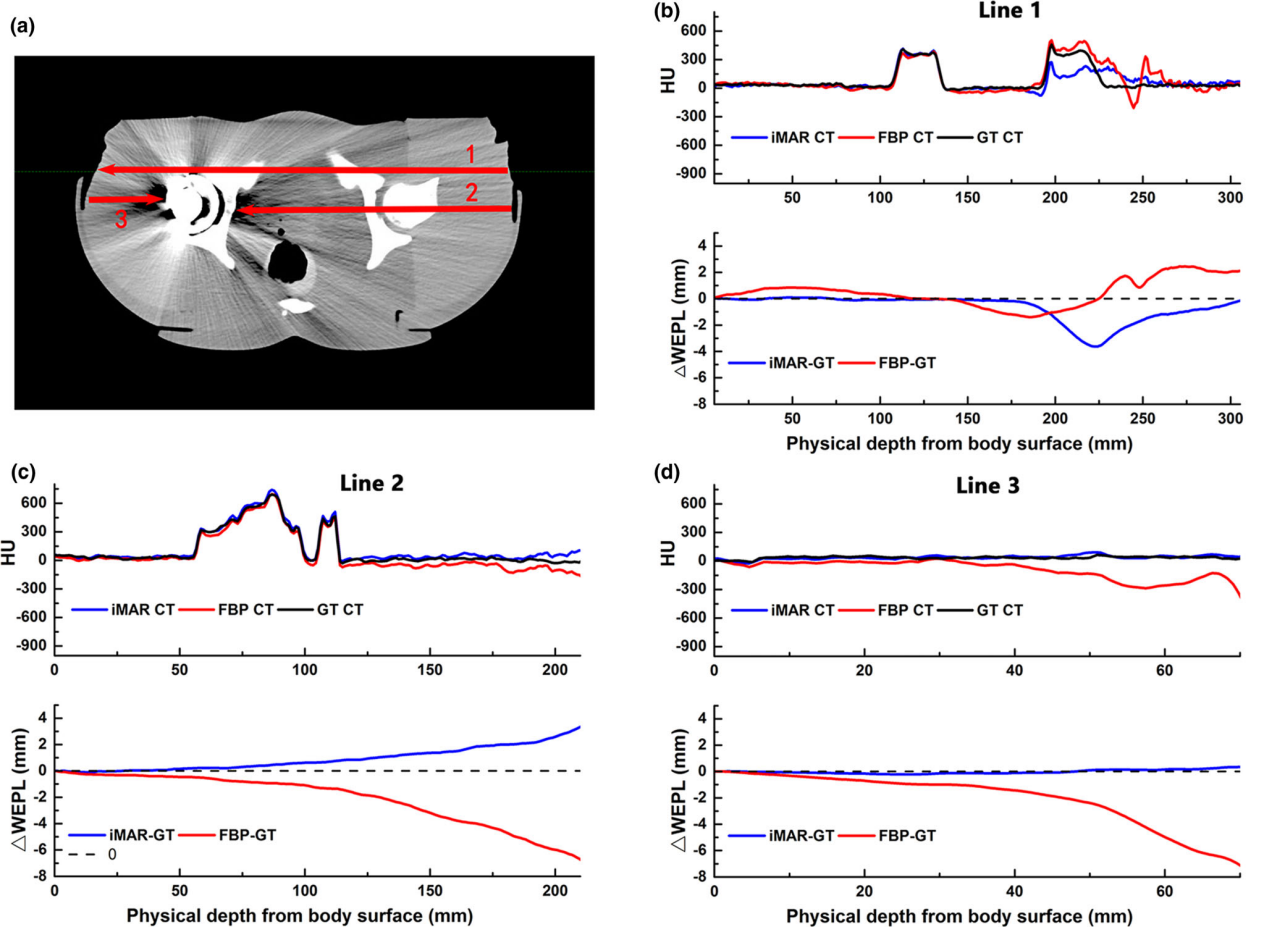
Profiles of CT numbers for the phantom with a unilateral hip prosthesis showing WEPL deviations along the three red lines are illustrated on FBP image (a). CT number profiles and WEPL deviations were compared between the FBP, iMAR and GT images along line 1, line 2 and line 3 (b‐d).

**Fig. 4:**
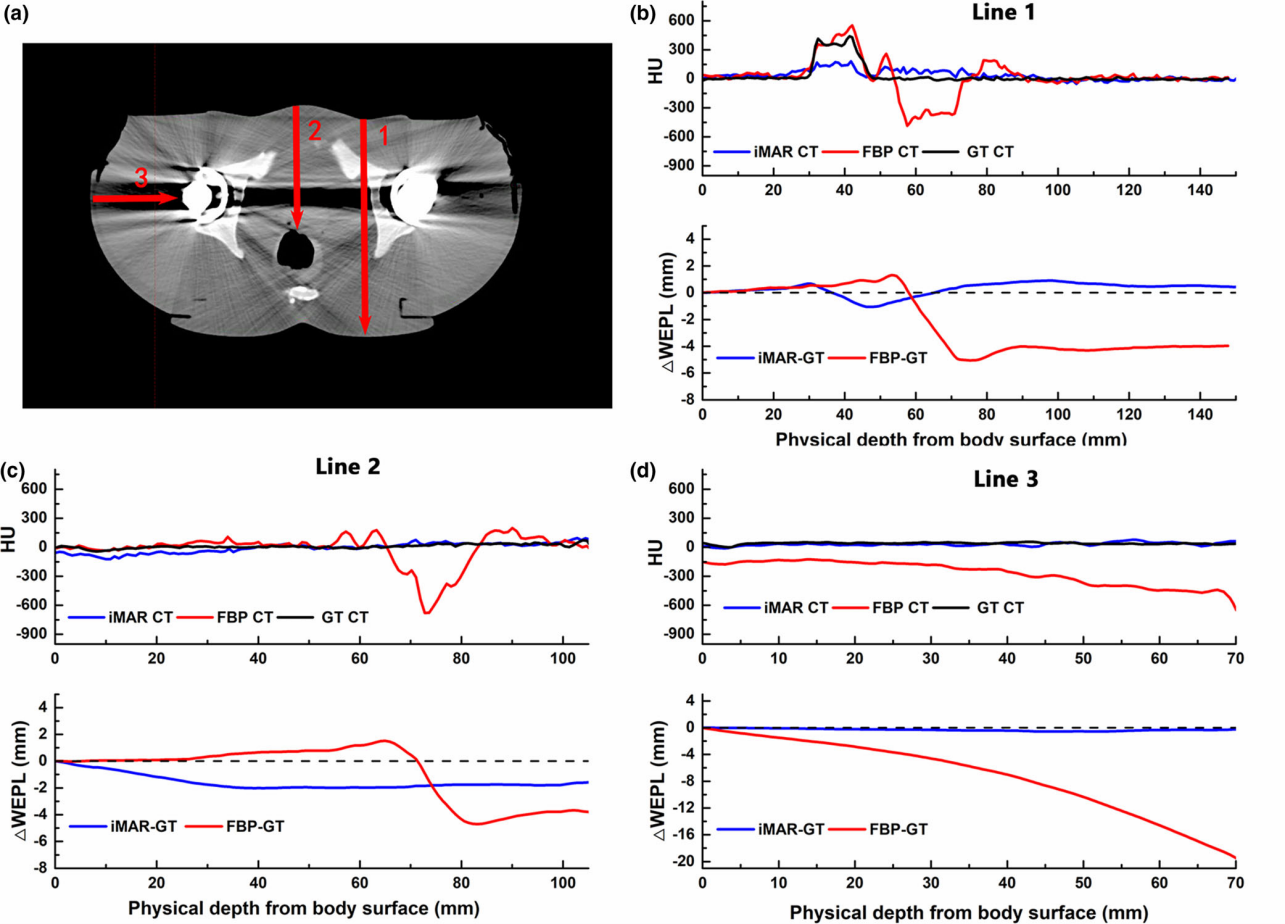
Profiles of CT numbers for the phantom with bilateral hip prosthesis showing WEPL deviations along the three red lines are illustrated on FBP image (a). CT number profiles and WEPL deviations were compared between the FBP, iMAR and GT images along line 1, line 2 and line 3 (b‐d).

WEPL deviations along several lines were compared between the FBP, iMAR, and GT images for the pelvic phantom with unilateral and bilateral hip‐prostheses, as indicated in Figs. 3 and 4, respectively. The WEPL spatially corresponded with the CT number profiles and took into account the beam path profiles according to the direction used to treat the clinical cases.

The iMAR algorithm improved the WEPL deviation at the distal of lines, with only some fluctuations in the middle of some lines. For the iMAR‐corrected images with a unilateral hip prosthesis, the maximum WEPL deviations were reduced from −8.1 to 0.4 mm, as shown in Fig. 3(d). The iMAR introduced bright artifacts near the periprosthetic bone resulted in a WEPL deviation of 3.3 mm at the distal path [Fig. 3(c)], which is still better than the −6.9 mm WEPL deviation obtained from the FBP images. With a failure to retrieve the CT numbers in periprosthetic bone material as in Fig. 3(b), the WEPL deviations in iMAR images increased to −3.6 mm in the midline. For the images with bilateral hip prostheses, the metal artifact was more conspicuous when compared with images with unilateral hip prosthesis, resulting in a larger WEPL deviation. iMAR succeeded in retrieving the CT number at the lowest CT value regions, with the WEPL deviation dramatically reduced from −19.1 to −0.4 mm [Fig 4(d)]. Although the WEPL deviation at the iMAR newly introduced artifact remained high at −2 mm [Fig 4(c)], it was still an improvement when compared with a WEPL of −4 mm obtained from the artifacts on the FBP images.

The iMAR algorithm had minor effects on the CT number of soft tissues located at the contralateral side of the hip prosthesis. The WEPL deviations were within 1 mm from the surface to the contralateral bony structures, as shown in Figs. 3(b) and 3(c).

#### 3.1.3 Dosimetric evaluation

Table 2 presents the DVH and gamma analysis for the FBP, iMAR, and GT images. In prostate cancer with unilateral hip prosthesis, the dose discrepancies in the PTV and critical organs between the FBP, iMAR, and GT images were less than 1%. Larger discrepancies were found in DVHs of PTVs and the gamma index of 2D planar dose between FBP and GT images for prostate cancer with bilateral hip prostheses and sarcoma cases with unilateral hip prosthesis. The use of iMAR improved the difference of DVH metrics of the target coverage within 1%, and the gamma index was more than 90% for both 3D global and 2D planar dose. For critical organs, however, the residual metal artifacts created by iMAR images in patients with bilateral hip prostheses resulted in uncertainties in the calculation of bladder doses with variations in the D_mean_ and V_D50 Gy(RBE)_ of −1.7% and 3.0%, respectively.

**Table 2:** Dose comparison of the optimized plans and recalculated plans on FBP, iMAR, and GT images.

		FBP	GT	Diff.	iMAR	GT	Diff.
(a) Prostate cancer with bilateral hip implants							
Gamma index	3D Global dose (γ < 1) (%)	95.3	/	/	97.2	/	/
	2D Planar dose (γ < 1) (%)	82.4	/	/	95.2	/	/
PTV	D_mean_ (%)	99.4	99.0	0.4	99.4	99.4	0.0
	D_V98%_ (%)	95.2	90.6	4.6	95.2	94.6	0.5
	V_D95%_ (%)	98.3	94.9	3.4	98.3	97.9	0.4
	D_1cc_ (%)	102.7	102.6	0.1	102.7	102.8	−0.1
Bladder	D_mean_ (%)	72.7	73.8	−1.1	72.4	74.1	−1.7
	V_D50 Gy(RBE)_ (%)	45.5	45.3	0.2	43.7	46.7	−3.0
Rectum	D_mean_ (%)	24.5	21.7	2.8	24.8	24.4	0.4
	V_D50 Gy(RBE)_ (%)	2.2	0.6	1.6	2.4	1.5	0.9
(b) Prostate cancer with unilateral hip prosthesis							
Gamma index	3D Global dose (γ < 1) (%)	98.0	/	/	97.9	/	/
	2D Planar dose (γ < 1) (%)	96.9	/	/	96.7	/	/
PTV	D_mean_ (%)	99.4	99.3	0.1	99.3	99.2	0.1
	D_V98%_ (%)	94.6	93.8	0.8	94.0	93.9	0.1
	V_D95%_ (%)	97.5	96.6	0.9	96.7	97.1	−0.4
	D_1cc_ (%)	101.2	101.5	−0.3	101.1	101.3	−0.2
Bladder	D_mean_ (%)	51.0	50.4	0.7	50.2	49.9	0.3
	V_D50 Gy(RBE)_ (%)	28.0	27.1	0.9	27.5	27.2	0.3
Rectum	D_mean_ (%)	22.9	22.5	0.3	22.4	23.0	−0.6
	V_D50 Gy(RBE)_ (%)	3.7	3.4	0.3	3.2	4.0	−0.8
(c) Sarcoma with unilateral hip prosthesis							
Gamma index	3D Global dose (γ < 1) (%)	95.6	/	/	96.1	/	/
	2D Planar dose (γ < 1) (%)	87.5	/	/	90.8	/	/
PTV	D_mean_ (%)	101.4	101.1	0.3	101.3	101.2	0.1
	D_V98%_ (%)	97.8	95.7	2.1	97.9	97.2	0.7
	V_D95%_ (%)	99.6	98.3	1.3	99.7	99.3	0.4
	D_1cc_ (%)	104.2	104.2	0.0	104.3	104.3	0.0

Note

D_V98%_: percentage of prescription dose covering 98% of the PTV; V_D95%_: percentage of PTV volume covered by 95% of prescription dose, D_1cc_: the percentage of the prescription dose received by 1cc of the PTV volume; D_mean_: mean dose; V_D50 Gy(RBE)_: the relative volume receiving 50 Gy (RBE).

**Fig. 5:**
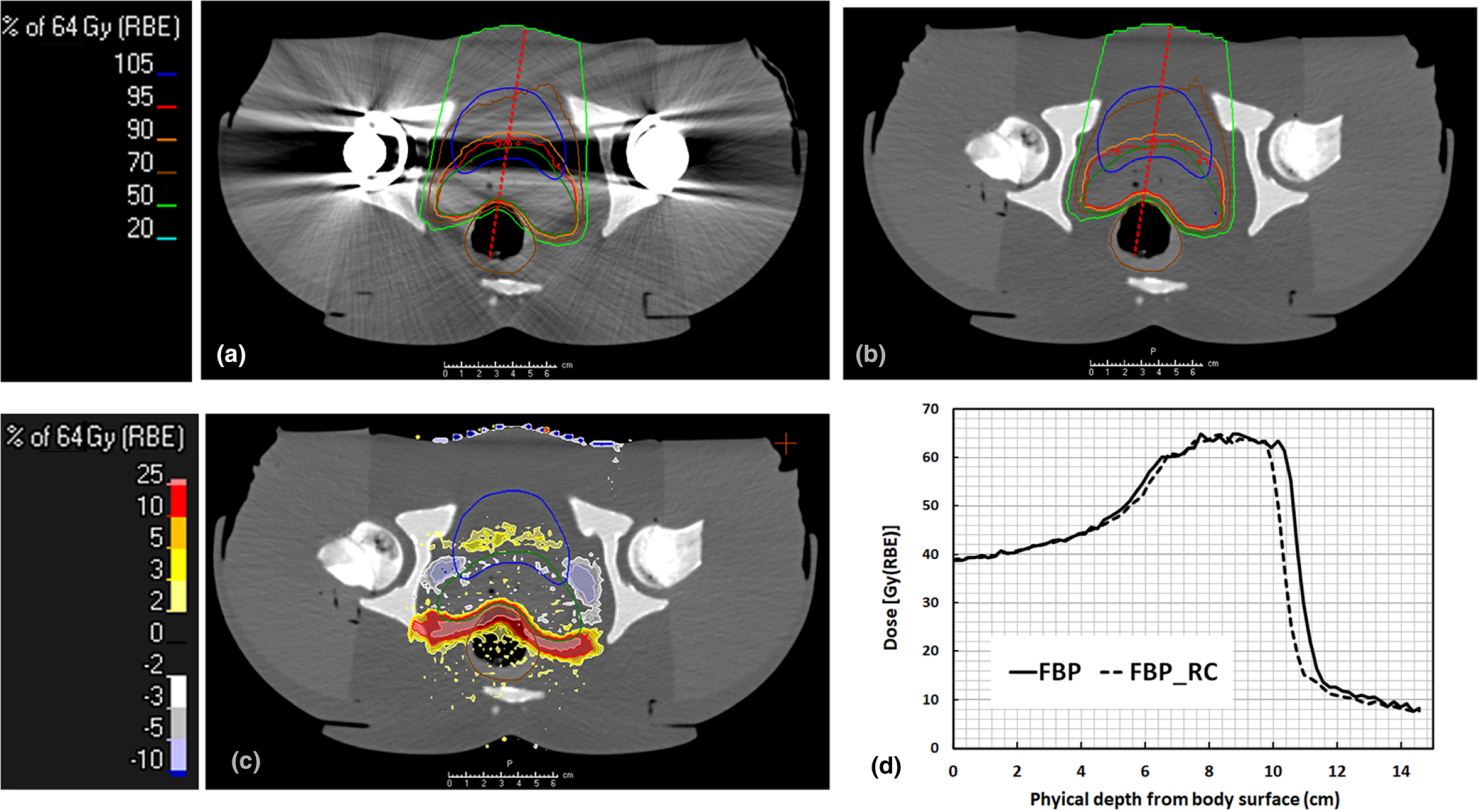
Dose distribution of treatment plans on phantom images with bilateral hip implants using FPB images (a) and the recalculated dose on the reference GT images (b). Image (c) illustrates the dose differences between the optimized dose minus the recalculated dose map on the GT images, and image (d) illustrates the dose profile through the most prominent artifact area indicated by the red dash lines on images (a) and (b)

**Fig. 6:**
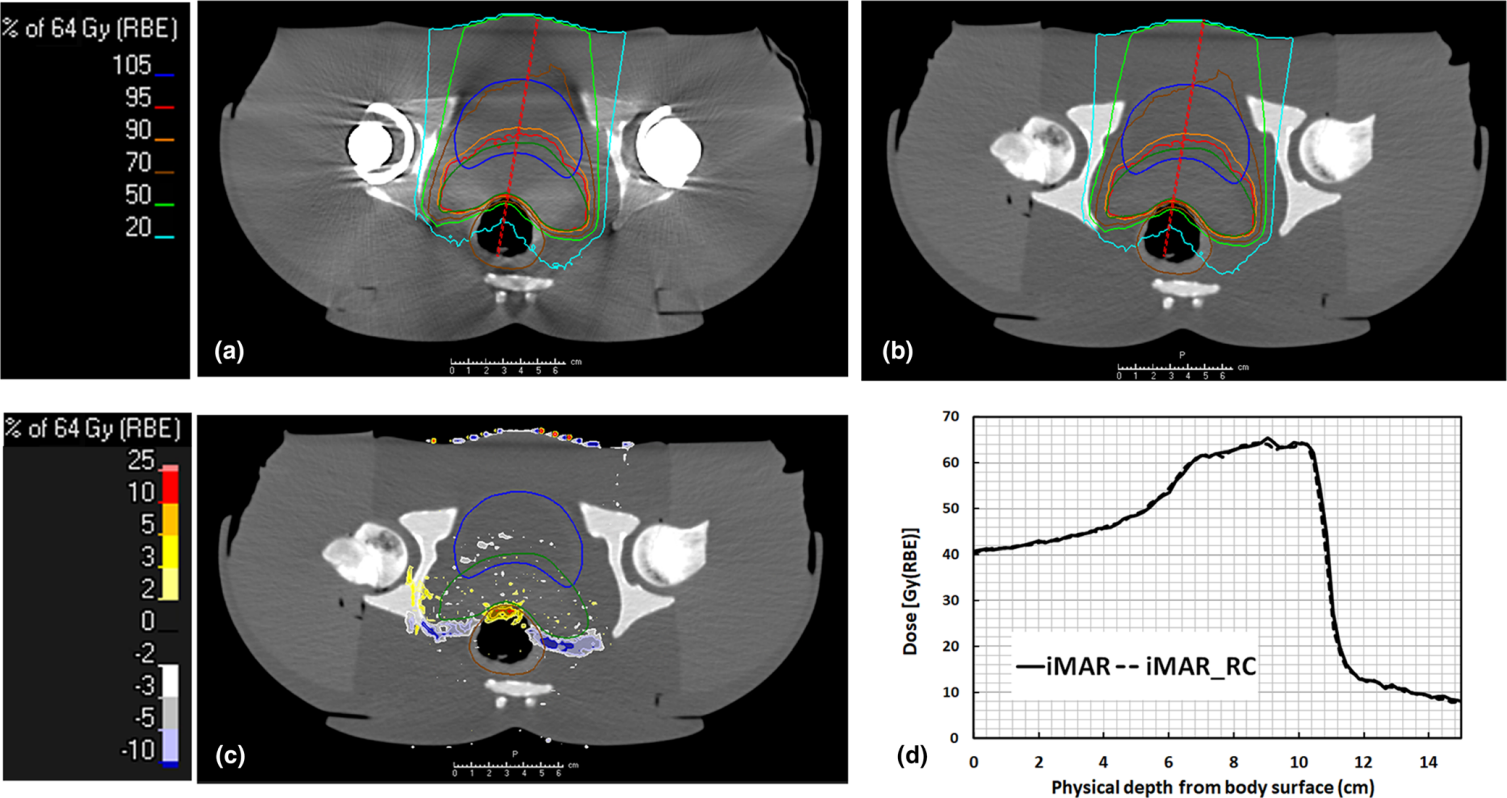
Dose distribution of treatment plans on phantom images with bilateral hip implants using iMAR images (a) and the recalculated dose on the reference GT images (b). Image (c) illustrates the dose differences between the optimized dose minus the recalculated dose map on the GT images, and image (d) illustrates the dose profile through the most prominent artifact area indicated by the red dash lines on images (a) and (b).

Figures 5 and 6 illustrate the optimized dose distribution of a treatment plan for prostate cancer with bilateral hip prostheses on the FBP, iMAR, and GT images, together with dose differences between them. When using iMAR, the dose differences were significantly improved but still existed. The dose profiles through critical organs are also illustrated in Figs. 5(d) and 6(d). The variations in the 90% dose line at the sampled area with serious artifacts decreased from −4.6 to −0.2 mm in iMAR images. However, differences of about 1 mm still existed at the lateral side of the rectum.

### 3.2 Patient study

#### 3.2.1 Quantitative image analysis

**Fig. 7:**
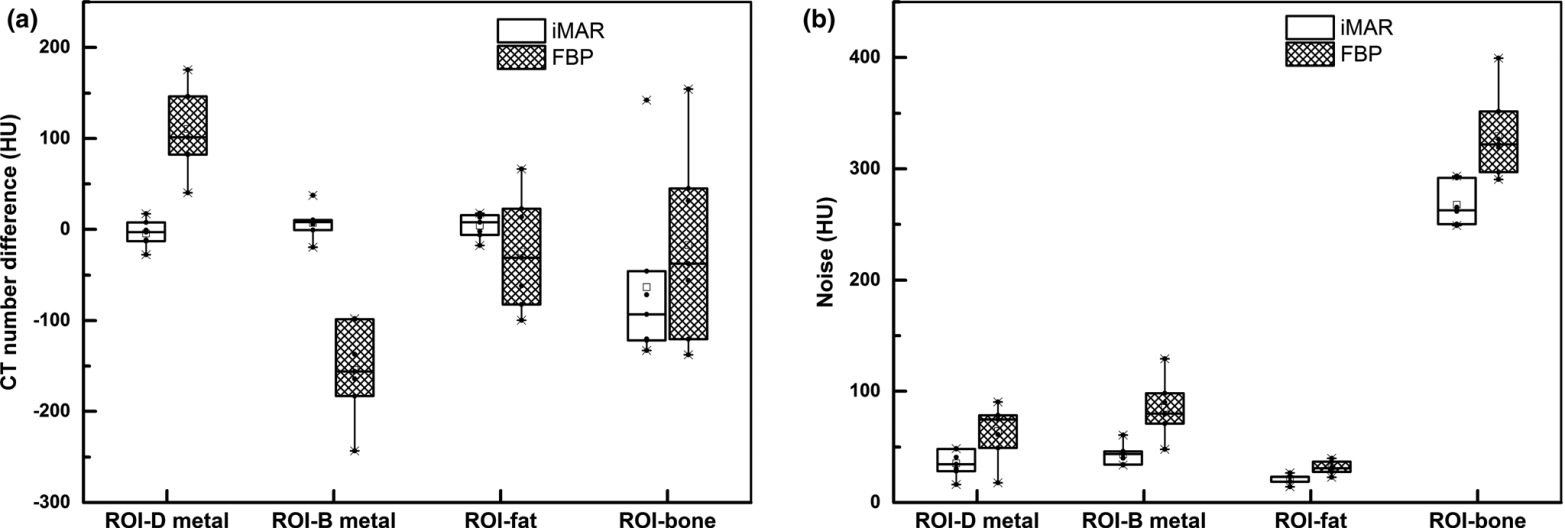
Box plots illustrating (a) CT number differences and (b) noise for the ROIs on the FBP and iMAR images compared with the reference ROIs.

As shown in Fig. 7(a), the iMAR algorithm reduced the CT number differences for most ROIs with the exception of ROI‐bone. For ROI‐DMetal, ROI‐BMetal, and ROI‐fat, the median values of CT number differences were improved from 101 HU (interquartile range, 82–146 HU) to −3 HU (interquartile range, −13 to −8 HU), −156 HU (interquartile range, −183 to −99 HU) to 8 HU (interquartile range, −1 to 10 HU), and −31 HU (interquartile range, −82 to 23 HU) to 8 HU (interquartile range, −6 to 18 HU), respectively. For ROI‐bone near metal hip prosthesis, even with iMAR correction, a large variation from the reference ROI in the CT number remained (median value −93 HU, interquartile range −122 to −46 HU). The noise was reduced for all ROIs on iMAR images [Fig. 7(b)].

#### 3.2.2 Dosimetric evaluation

**Fig. 8:**
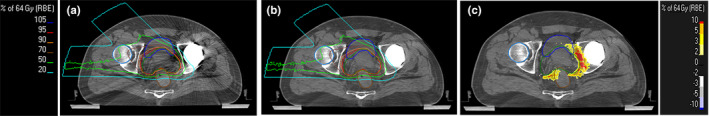
Dose distribution of treatment plans for a prostate cancer case with a unilateral hip prosthesis for the FBP images (a), recalculated plans on iMAR images (b), and the dose differences between the optimized dose and the recalculated dose map on iMAR image (c).

Figure 8 shows the dose distribution on the FBP and iMAR images for prostate cancer with unilateral hip prosthesis, together with their dose differences. Table 3 presents the statistical data of target coverage, critical organs, and gamma analysis on FBP and iMAR images.

**Table 3:** Dose comparison of treatment plan optimized on FBP images and recalculated on iMAR images for all patient cases.

No.	Indication	PTV						Bladder				Rectum				Gamma analysis	
		Mean (%)		D_V98%_ (%)		V_D95%_ (%)		Mean (%)		V_D50 Gy(RBE)_ (%)		Mean (%)		V_D50 Gy(RBE)_ (%)		3D Global dose (%)	2D planar dose (%)
		FBP	iMAR	FBP	iMAR	FBP	iMAR	FBP	iMAR	FBP	iMAR	FBP	iMAR	FBP	iMAR		
1	Osteosarcoma	99.6	98.9	97.6	84.2	99.8	94.1	28.0	27.7	11.0	10.5	7.5	7.3	0.1	0.0	89.2	71.2
2	Osteosarcoma	99.9	100.0	97.3	96.2	99.9	99.9	1.5	1.4	0.0	0.0	38.8	38.9	19.4	19.0	97.2	93.9
3	Chondrosarcoma	99.8	99.7	96.6	96.2	100	99.6	8.9	8.8	0.9	0.9	19.9	20.1	0.0	0.0	96.1	88.9
4	Chondrosarcoma	99.9	99.9	98.0	97.8	100.0	100.0	5.1	5.2	0.8	0.8	1.2	1.2	0.0	0.0	97.5	94.2
5	Prostate cancer	99.7	99.5	95.6	95.3	98.5	98.4	31.6	31.6	14.1	14.1	42.2	42.1	21.5	21.3	98.0	95.2
6	Prostate cancer	99.8	99.8	95.0	94.1	98.0	97.5	37.6	37.9	16.8	16.9	31.6	31.1	14.2	13.9	99.6	97.8
7	Prostate cancer	101.3	100.9	97.4	96.1	99.5	98.7	39.2	39.1	18.6	18.9	37.2	36.8	14.4	13.9	97.6	95.4

For prostate cancer, the results showed a slight decrease in target coverage following dose recalculated on the iMAR images with a maximum D_V98%_ reduction of 1.3% for case 7. Most of the dose differences were located at the dark streak area of metal artifacts. Gamma index was over 95% for both 3D global and 2D planar dose. For sarcoma cases, the treatment site was located very close to the prosthesis and hence directly affected by the metal artifacts, especially dark streaks. Large discrepancies in the PTV dose were found in case 1, with a maximum D_V98%_ deviation of 13.5%. Consistently, the gamma index was less than 90% for both 3D global and 2D planar dose for case 1. For all other cases, the differences in the DVH parameters for the PTVs and critical organs were all below 1.1%, and the gamma index was above 90%, except the third case with a 2D planar gamma index of 88.9%.

## 4 DISCUSSION

iMAR reduced the metal artifacts, thus improved the retrieval of CT numbers on both phantom and patient images. Our findings were in line with previous studies.^14^, ^25^ Moreover, we performed dosimetric investigations utilizing iMAR images for pelvic cancer with hip prostheses. iMAR potentially improves the dose calculation accuracy for carbon ion treatment planning.

In the phantom study, the WEPL comparisons showed that iMAR improved the accuracy of beam range analysis and had a minor effect on the CT number for tissue located far away from the metal implant. On the images with a unilateral hip prosthesis, the iMAR algorithm resulted in a similar WEPL on the GT images on the contralateral side of the prosthesis. However, it was not possible to remove all WEPL deviations with iMAR. The residual deviations were mainly located at the periprosthetic bone and its surrounding soft tissue. Besides, the artifacts in the pelvic phantom were larger, leading to a WEPL deviation of several millimeters, which could potentially affect the accuracy of the beam range calculation. For unilateral hip prosthesis, oblique and contralateral beams, avoiding new artifacts, are used to reduce inaccuracies in the dose calculations caused by metal artifacts. For bilateral hip prostheses, these artifacts accumulated between the periprosthetic bones, and therefore, it was not possible to avoid these artifacts by modifying the limited beam angles.

For the dosimetric study on the phantom images, iMAR improved the large dose discrepancies in DVHs of PTV and gamma index of sarcoma and prostate cancer with bilateral hip prostheses between FBP and GT images. However, iMAR also introduced new artifacts, which reduced the dose calculation accuracy to critical organs for bilateral hip prostheses. This implies that iMAR may guarantee adequate PTV dose coverage, but it may potentially reduce the accuracy of the dose calculations for critical organs in prostate cancer patients with bilateral hip prostheses. The overriding of serious iMAR introduced artifacts might be needed for cases with bilateral hip prostheses.

The patient study was conducted on prostate and sarcoma cases with a unilateral hip prosthesis. For prostate cancer, D_V98%_ and V_D95%_ showed a small decrease in recalculated dose on iMAR images. The difference was small and mainly located at the edge of the PTV close to the implants, within the dark streaks caused by the metal artifacts on FBP images. iMAR corrected these artifacts according to the density of the similar surrounding tissue. Thus, dose distribution on the iMAR image could be closer to the real situation. For sarcoma cases, the discrepancies of DVHs in PTVs were large in some cases. The functionality of the iMAR correction was confirmed by manually correcting the FBP images for this case (Supporting Information S1). The optimized dose distribution on the manually corrected FBP images and the recalculated dose distribution on iMAR images were similar. Though some extra uncertainties could be brought in, manual correction is still considered to be the strategy for clinical treatment planning with metal implants.^10^, ^26^ Therefore, the comparable dose distribution implies that the dose calculated on iMAR images is a suitable alternative for dose calculation in patients with implants.

In order to reduce dose calculation errors caused by metal artifacts, as an example in Rana's study of proton planning for prostate cancer patients with a metalic hip prosthesis, 16 artifacts and structures were manually segmented and overriden with the appropriate RLSPs in metal artifact contained planning CT images.^10^ The use of iMAR could potentially bypass this laborious and subjective manual segmentation process while still ensuring the correct dose distribution. However, for periprosthetic bony structure, the CT numbers restored by iMAR were less accurate in both phantom and patient studies. Hence, in the cases whereby the beam passes through the periprosthetic bony structure, the manual density overriding method may be more accurate.

In our study, we focused on the metal artifacts in the soft tissue and bone, but not in the metal. The metal implants were placed either at the distal edge of the beam or outside of the beam path, which would not affect the calculation of WEPL and thus dose distribution. Therefore, the results of this study can be applied to proton therapy since the differences in the RLSP between proton and carbon ions of the same range are less than 1% in human tissue except for lung.^27^ However, if the beam passes through the metal, the attenuation, interface effects, and neutron production vary between different kinds of particles. Therefore, future studies should focus on evaluating dose calculation inaccuracies for cases whereby the beam passes through the metal implant using different types of particles.

The study has some limitations that have to be acknowledged. Only one type of hip prostheses with Co‐Cr‐Mo and Ti alloy was evaluated. The physical properties of metal implants, such as shape, size, and material composition, may have an important impact on the generated artifacts and accuracy of the dose calculations.^1^ Therefore, more research is required to evaluate the impact of iMAR on dosimetry for different hip prostheses. Furthermore, dosimetric evaluation for bilateral hip replacements was only performed on a phantom as patient cases are rare, highlighting the need for further studies to evaluate the dose impact on actual studies.

## 5 CONCLUSION

iMAR is an effective tool that can be used to reduce metal artifacts and improve the calculation accuracy for particle therapy of pelvic cancer with unilateral hip prosthesis. This implies that iMAR images can be implemented in the treatment planning of particle therapy to replace the traditional method of manual overriding. However, it should be used with caution due to the residue and new artifacts generated by iMAR, especially in periprosthetic bone and patients with bilateral hip prostheses.

## CONFLICT OF INTEREST

The authors report no conflict of interest.

## AUTHOR CONTRIBUTIONS

Jingfang Zhao, Weiwei Wang, Kambiz Shahnaz and Qing Zhang contributed to concept of the study.

Jingfang Zhao, Ping Li and Xianwei Wu performed data acquisition and analysis.

Kambiz Shahnaz, Jingfang Mao, and Qing Zhang supervised the study.

Jingfang Zhao drafted the manuscript.

Jingfang Zhao, Jingfang Mao, Weiwei Wang, Kambiz Shahnaz, Ping Li, and Qing Zhang reviewed and edited the manuscript.

Jingfang Zhao and Jingfang Mao contributed to the funding acquisition.

## Supporting information

Fig S1 Dose distribution of treatment plans on uncorrected and corrected FBP images and 14 iMAR images with artefacts for Patient 1 with unilateral hip implants, together with their 15 differences map to iMAR images. (a) The optimized dose of treatment plan on artefact‐16 uncorrected images. (b) The recalculated dose of treatment plan on iMAR images. (c) 17 Differences between optimized dose on FBP images with uncorrected artefacts and 18 recalculated dose map of iMAR images. (d) The optimized dose of treatment plan on artefact‐19 corrected images. (e) The recalculated dose of treatment plan on iMAR images. (f) Differences 20 between optimized dose on FBP images with corrected artefacts and recalculated dose map 21 of iMAR images.

Table S1 Dose comparison of optimized plan on artefact‐uncorrected/corrected images and 2 recalculated plan on iMAR images for Patient 1 with unilateral hip implants.

## Data Availability

The data that support the findings of this study are available from the corresponding author upon reasonable request.
