# Supplementary figures and images for: Dosimetric impact of using a commercial metal artifact reduction tool in carbon ion therapy in patients with hip prostheses
Source: J Appl Clin Med Phys. 2021 Jun 23;22(7):224–34. doi: 10.1002/acm2.13314 (PMC8292709; doi:10.1002/acm2.13314)

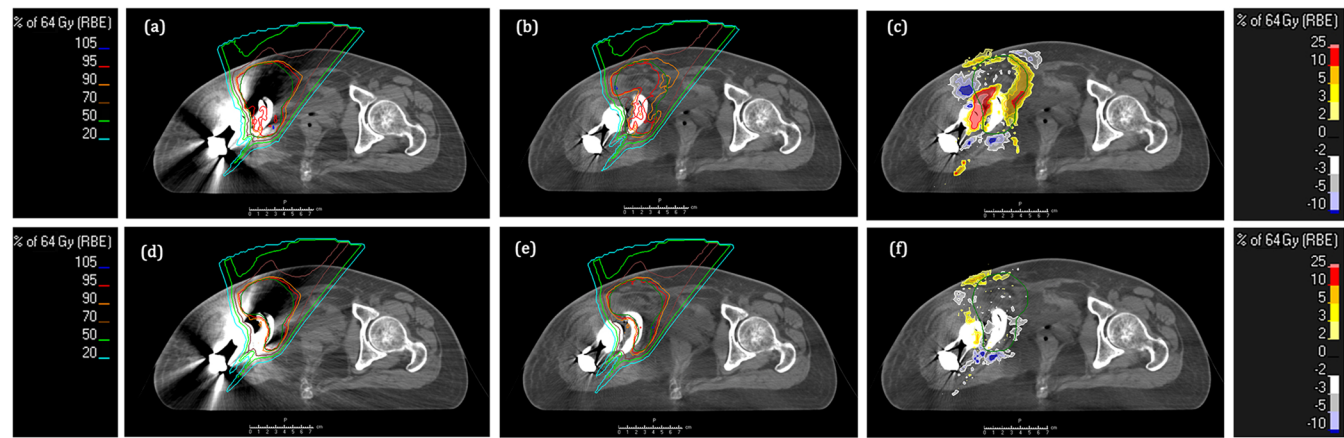

Supplement: Supplementary file 1 — Fig S1 Dose distribution of treatment plans on uncorrected and corrected FBP images and 14 iMAR images with artefacts for Patient 1 with unilateral hip implants, together with their 15 differences map to iMAR images. (a) The optimized dose of treatment plan on artefact‐16 uncorrected images. (b) The recalculated dose of treatment plan on iMAR images. (c) 17 Differences between optimized dose on FBP images with uncorrected artefacts and 18 recalculated dose map of iMAR images. (d) The optimized dose of treatment plan on artefact‐19 corrected images. (e) The recalculated dose of treatment plan on iMAR images. (f) Differences 20 between optimized dose on FBP images with corrected artefacts and recalculated dose map 21 of iMAR images. [file ACM2-22-224-s002.pdf]
