# Supplementary material for: Dosimetric impact of using a commercial metal artifact reduction tool in carbon ion therapy in patients with hip prostheses
Source: J Appl Clin Med Phys. 2021 Jun 23;22(7):224–34. doi: 10.1002/acm2.13314 (PMC8292709; doi:10.1002/acm2.13314)
Supplement: Supplementary file 2 — Table S1 Dose comparison of optimized plan on artefact‐uncorrected/corrected images and 2 recalculated plan on iMAR images for Patient 1 with unilateral hip implants. [file ACM2-22-224-s001.pdf]

**Supplemental material Ⅰ**

Table 1 Dose comparison of optimized plan on artefact-uncorrected/corrected images and recalculated plan on iMAR images for Patient 1 with unilateral hip implants

| | | FBP Uncorr. | iMAR | Diff. | FBP corr. | iMAR | Diff. |
|---|---|---|---|---|---|---|---|
| Patient 1: Osteosarcoma | | | | | | | |
| Gamma index | 3D Global dose (γ<1) (%) | 89.2 | / | / | 95.4 | / | / |
| | 2D Planar dose (γ<1) (%) | 71.2 | / | / | 93.4 | / | / |
| PTV | $D_{mean}$ (%) | 99.6 | 98.9 | 0.7 | 99.6 | 99.6 | 0 |
| | $D_{V98\%}$ (%) | 97.6 | 84.2 | 13.5 | 98.0 | 97.6 | 0.4 |
| | $V_{D95\%}$ (%) | 99.8 | 94.1 | 5.7 | 100.0 | 100.0 | 0 |
| Bladder | $D_{mean}$ (%) | 28.0 | 27.7 | 0.3 | 28.5 | 28.6 | -0.1 |
| | $V_{D50Gy(RBE)}$ (%) | 11.0 | 10.5 | 0.5 | 11.3 | 11.4 | -0.1 |
| Rectum | $D_{mean}$ (%) | 7.5 | 7.3 | 0.2 | 7.8 | 7.7 | 0.1 |
| | $V_{D50Gy(RBE)}$ (%) | 0.1 | 0.0 | 0.1 | 0.1 | 0.0 | 0.1 |
